# Does the Warren Shunt Correct Hypersplenism?

**DOI:** 10.1155/1990/30752

**Published:** 1990

**Authors:** J. Peter A. Lodge, Andrew I. D. Mavor, Geoffrey R. Giles

**Affiliations:** 1 University Department of Surgery St James's University Hospital Leeds United Kingdom; 2 University Department of Surgery Clinical Sciences Building St James's University Hospital Beckett Street Leeds LS9 7TF United Kingdom

## Abstract

It has been suggested that patients with bleeding varices and hypersplenism will show significant improvements in leucocyte and platelet counts following distal splenorenal (Warren) shunt surgery. Whilst this may be true in the short term, this report shows that in the long term hypersplenism is not relieved, whereas the lienorenal shunt is associated with a return of normal haematological values.


*HPB Surgery*, 1990, Vol. 2, pp. 41–49
Reprints available directly from the publisher
Photocopying permitted by license only
© 1990 Harwood Academic Publishers GmbH
Printed in the United Kingdom

### DOES THE WARREN SHUNT CORRECT HYPERSPLENISM?

J. PETER A. LODGE, ANDREW I. D. MAVOR and GEOFFREY R. GILES

*University Department of Surgery, St James's University Hospital, Leeds, U.K.*

*(Received: 12 May 1988; in final form 27 April 1989)*

It has been suggested that patients with bleeding varices and hypersplenism will show significant improvements in leucocyte and platelet counts following distal splenorenal (Warren) shunt surgery. Whilst this may be true in the short term, this report shows that in the long term hypersplenism is not relieved, whereas the lienorenal shunt is associated with a return of normal haematological values.

KEY WORDS: Hypersplenism, distal splenorenal (Warren) shunt (DSRS), lienorenal shunt (LRS), portosystemic shunt (PSS)

### INTRODUCTION

Hypersplenism may be present in 55% of patients with portal hypertension[1]. It produces a pancytopaenia and is usually associated with an enlarged spleen. It is the result of hyperplasia of the reticulo-endothelial system within the spleen and not merely a consequence of the portal hypertension and venous congestion. The leucopaenia, which affects mainly the polymorph population, is usually of the order of 2.5– $3.0 \times 10^9/l$. Thrombocytopaenia is not usually severe; quite commonly there is a mild anaemia with a reduced red cell count. It is uncertain whether these changes result in the development of symptoms. However, in patients who have bled from varices the lowered platelet count may be a factor in the delay of spontaneous cessation of the bleeding, and a lowered polymorph count may predispose to infection.

Hypersplenism in association with portal hypertension may clearly be dealt with by the removal of the spleen and the construction of a lienorenal shunt. It is questionable, however, whether the simple relief of pressure in the splenic venous system is sufficient to correct the haematological changes. This study was performed to examine the course of patients undergoing shunt surgery for actively bleeding oesophageal varices and compared the haematological changes following: a) a lienorenal shunt [LRS] (relief of portal hypertension and splenectomy); b) a central portosystemic shunt [portacaval or mesocaval shunt - PSS] (relief of portal hypertension leaving the spleen in situ); and c) a distal splenorenal (Warren) shunt [DSRS] (selective decompression of the spleen).

*Correspondence to: Mr J. P. A. Lodge, University Department of Surgery, Clinical Sciences Building, St. James's University Hospital, Beckett Street, Leeds LS9 7TF.

### PATIENTS AND METHODS

In this retrospective study we analysed patients undergoing shunt surgery for actively bleeding oesophageal varices over a 15 year period (1971–1986) and followed their leucocyte and platelet counts over a follow up period of at least one year. During this period 116 shunt procedures were performed: 28 LRS, 35 PSS, and 53 DSRS. A summary of diagnoses is shown in Table 1. White blood cell and platelet counts were recorded at admission, discharge from hospital (average hospital stay 29 days) and at one year post-operation. Hypersplenism was defined as leucopaenia (white cell count less than $5 \times 10^9$/l) and/or thrombocytopaenia (platelet count less than $140 \times 10^{12}$/l), figures used routinely by our haematology laboratory and based on studies of all patients entering Yorkshire hospitals or donating blood over a number of years. Elevation of counts to above these figures qualified as relief of hypersplenism.

### RESULTS

Twenty-three of the 53 DSRS, 17 of the 28 LRS and 13 of the 35 PSS patients survived with adequate follow up for inclusion in this study. Preoperatively 14 of the 23 DSRS, 16 of the 17 LRS and 3 of the 13 PSS patients were defined as hypersplenic. The diagnoses of these patients are shown in brackets in Table 1.

**Table 1** Diagnosis

| | *Hypersplenic* | | | *Normosplenic* | | |
|---|---|---|---|---|---|---|
| | DSRS | LRS | PSS | DSRS | LRS | PSS |
| Alcoholic Cirrhosis | 8 (3) | 0 | 5 | 8 | 2 | 3 |
| Other forms of Cirrhosis | 17 (9) | 8 (8) | 6 (2) | 18 | 5 | 12 |
| Non-Cirrhotic Portal Hypertension | 2 (2) | 8 (8) | 2 (1) | 0 | 5 | 7 |

Figures in brackets: Hypersplenic patients with adequate follow-up.

To determine the effectiveness of selective splenic decompression the white cell and platelet counts of the hypersplenic DSRS and LRS patients were compared (Figures 1 and 2). All patients undergoing LRS had platelet counts at or above normal at discharge and at one year; 3 had a relative leucopaenia at discharge but all white cell counts were normal by one year. In contrast, all but 4 patients with DSRS showed improvement of leucocyte and platelet counts at discharge, but by the end of one year only 3 patients could be classified as having normal counts. Furthermore, analysis of the haematological parameters in the 9 DSRS patients who were normosplenic preoperatively showed that only one patient remained normosplenic by one year, the other 8 being classified as hypersplenic by their white cell count (2 patients), platelet count (3 patients) or both (3 patients) (Figure 3).

In the PSS group the 3 patients classified as hypersplenic preoperatively remained so, although in all 3 the thrombocytopaenia had resolved by discharge and this change was maintained at one year in 2. None of the normosplenic PSS patients became hypersplenic (Figure 4).

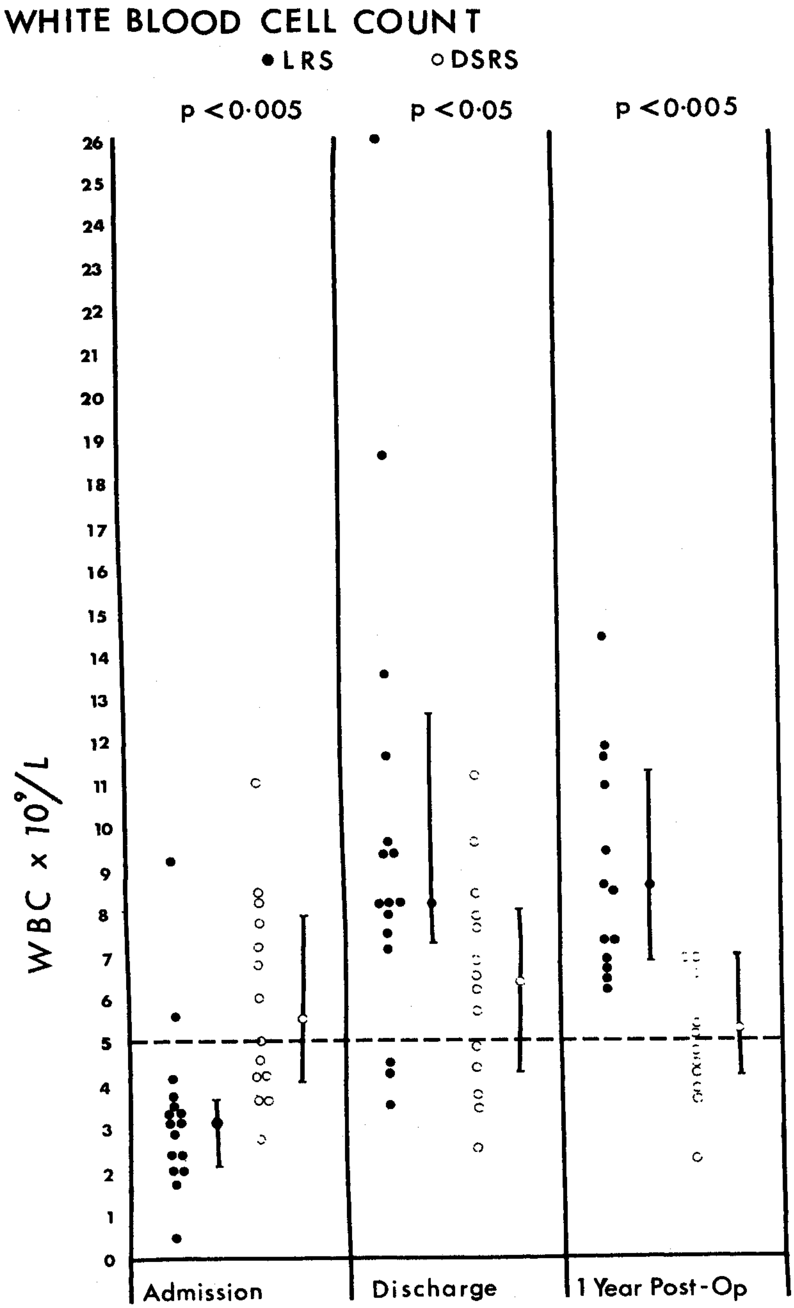

**Figure 1** White blood cell counts in DSRS and LRS patients who were hypersplenic preoperatively.

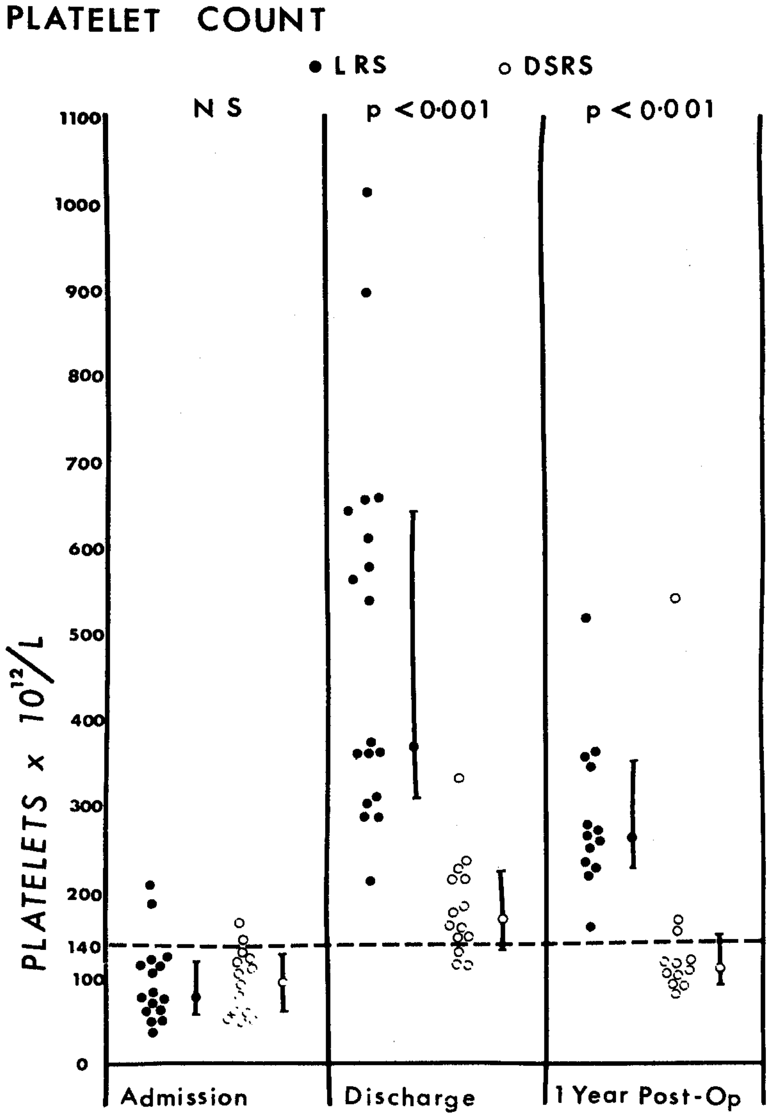

**Figure 2** Platelet counts in DSRS and LRS patients who were hypersplenic preoperatively.

(Figure 1 and 2: Median and interquartile ranges emphasised and statistical differences between the two shunt groups noted at the top of each column. N.S. – not significant; statistical analysis by Mann Whitney U test).

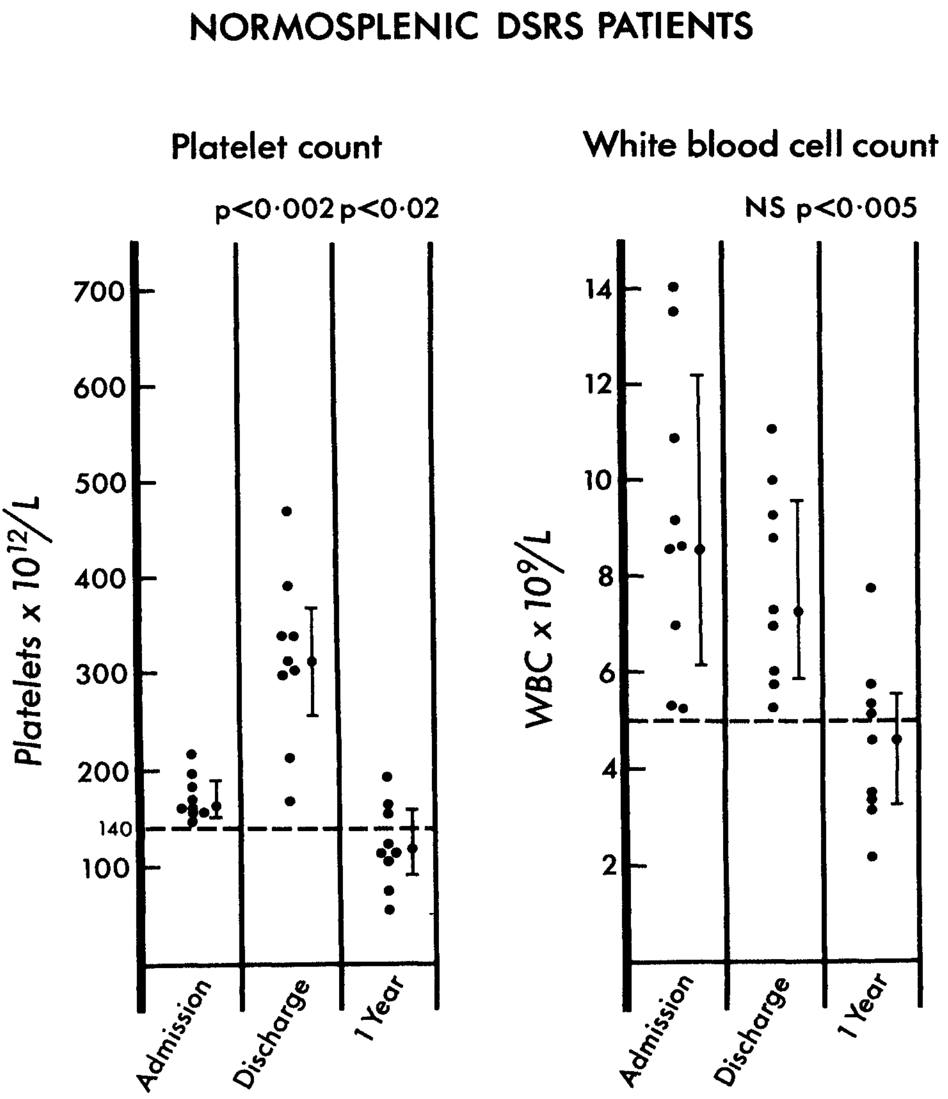

**Figure 3** White blood cell and platelet counts in DSRS patients who were normosplenic preoperatively.

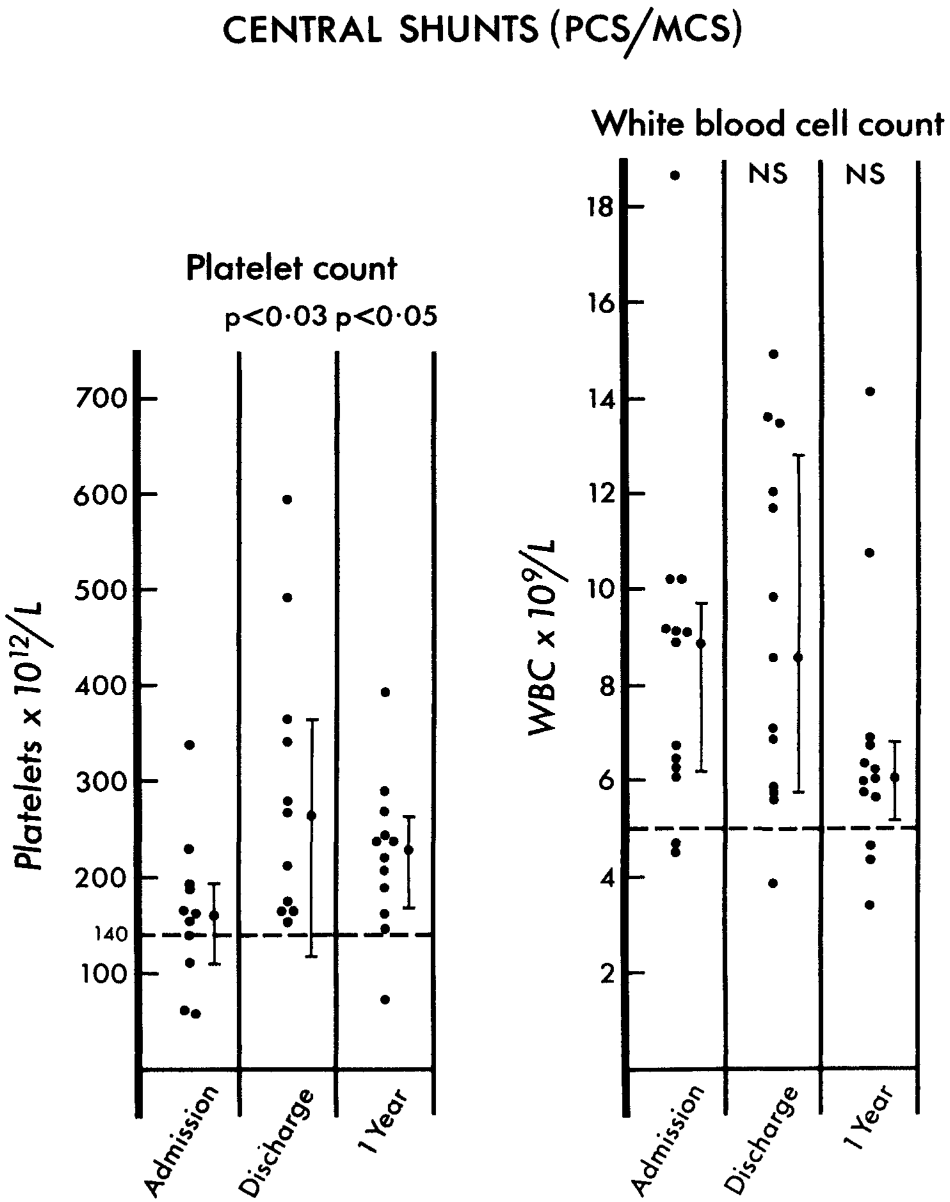

**Figure 4** White blood cell and platelet counts in PSS patients. (PCS – portacaval shunt; MCS – mesocaval shunt).

(Figure 3 and 4: Median and interquartile ranges emphasised. Mann Whitney U test used to compare discharge and one year values with preoperative figures. N.S. – not significant).

### DISCUSSION

Hypersplenism has not been judged to be a contraindication to distal splenorenal (Warren) shunt surgery as in the short term significant improvements in leucopaenia and thrombocytopaenia have been observed[1-6], and this shunt remains the surgical treatment of choice for variceal bleeding in several major institutions[6,7,8]. However, these studies looked only at short term follow-up in the majority of patients and they failed to take into account the natural fluctuation in the degree of hypersplenism that may be present at any one time[9]. A recent comprehensive review by Henderson has debated the advantages of selective shunting procedures[10]. Our own work has shown no significant differences in short or long term results of the DSRS and LRS in a non- randomised retrospective study[11].

The recurrence of thrombocytopaenia has been proposed as an indicator of shunt thrombosis, but there is no definite evidence to support this. This complication affected only one of the hypersplenic DSRS patients included in this study. A recent case report of a patient who redeveloped hypersplenism following a DSRS when his condition became complicated by cardiomyopathy and congestive heart failure implicated a recurrence of splenic congestion as the cause[12].

Our results suggest that although decompression of the spleen may be accomplished by the DSRS, the deleterious effect of hypersplenism on peripheral blood continues as in the non-operated patient; and splenectomy, as in the LRS, should be considered in the hypersplenic patient with bleeding varices.

### *References*

1. Ferrara, J., Ellison, C., Martin, E.W., Cooperman, M. (1979) Correction of hypersplenism following distal splenorenal shunt. *Surg.*, **86**, 570–3.
2. Van, J., Simert, G., Hanson, J.A., Thylen, U., Bengmark, S. (1977) Results of a modified distal spleno-renal shunt for portal hypertension. *Ann. Surg.* **185**, 224–8.
3. Soper, N.J., Rikkers, L.F. (1982) Effect of operations for variceal haemorrhage on hypersplenism. *Am. J. Surg.*, **144**, 700- 703.
4. El-Khishen, M.A., Henderson, J.M., Millikan, W.J. Jr, Kutner, M.H., Warren, W.D. (1985) Splenectomy is contraindicated for thrombocytopenia secondary to portal hypertension. *Surg. Gyn. Obs.*, **160**, 233–8.
5. Hutson, D.G., Zeppa, R., Levi, J.U., Schiff, E.R., Livingstone, A.S., Fink, P. (1977) The effect of the distal splenorenal shunt on hypersplenism. *Ann. Surg.*, **185**, 605–12.
6. Marni, A., Trojsi, C., Belli, L. (1981) Distal splenorenal shunt: Haemodynamic advantage over total shunt and influence on clinical status, hepatic function and hypersplenism. *Am. J. Surg*, **142**, 372–6.
7. Rikkers, L.F., Rudman, D., Galambos, J.T., Fulenwider, J.T., Millikan, W.J., Kutner, M., Smith, R.B., Salam, A.A., Jones, P.J., Warren, W.D. (1978) A randomised controlled trial of the distal splenorenal shunt. *Ann. Surg.*, **188**, 271–82.
8. Adson, M.A., van Heerden, J.A., Ilstrup, D.M. (1984) The distal splenorenal shunt. *Arch. Sur.*, **119**, 609–14.
9. Mutchnick, M.G., Lerner, E., Conn, H.O. (1980) Effect of portacaval anastomsis on hypersplenism. Digestive Diseases and Sciences, 25, 929–38.
10. Henderson, J.M. (1986) Variceal bleeding: Which shunt? (Editorial). *Gastroenterology*, **91** 1021–3.
11. Lodge, J.P.A., Mavor, A.I.D., Giles, G.R. (1989) Distal splenorenal versus lienorenal shunt for acute variceal haemorrhage: is the selective shunt an advance? Journal of the Royal College of Surgeons of Edinburgh. **34**, 59–62.
12. Garner, W.L., Vaccaro, P.S., Carey, L.C. (1986) Recurrent hypersplenism caused by alcoholic cardiomyopathy after distal splenorenal shunt. *Surg.*, **99**, 514–6.

*(Accepted by S. Bengmark)*

### INVITED COMMENTARY

Several studies including the one by Lodge and associates[1] in this issue of HPB Surgery have shown that hypersplenism is commonly associated with portal hypertension. Although splenic congestion may be one causative factor, the severity of hypersplenism appears to be more closely related to the degree of portal-systemic shunting than to the magnitude of portal pressure[2]. One possible mechanism for the hyperplasia of the splenic component of the reticuloendothelial system is antigenic stimulation by gut-derived antigens that escape Kupffer cell sequestration via intra- or extra-hepatic portal-systemic shunt pathways[3]. Spleen size and severity of hypersplenism tend to be greater in nonalcoholic cirrhotics than in patients with alcoholic cirrhosis[2]. The reasons for the difference between alcoholics and nonalcoholics is not clear.

Several investigations have addressed the issue of how effective nonselective and selective shunts are in relieving preoperative hypersplenism. Most studies[4–7] have shown a beneficial effect of portal decompression on hypersplenism and the improved white blood cell and platelet counts have persisted into the late postoperative interval in some of these series.[4,5] One investigation has shown no consistent influence of the portacaval shunt on hypersplenism[8]. The negative results of this study may be due to the predominance of alcoholic cirrhotics who may suppress platelet, red cell and white cell production via the toxic effects of alcohol on the bone marrow.

However, the most important point concerning the study by Lodge and associates is that hypersplenism secondary to portal hypertension is almost never clinically significant (platelet count less than 25,000/$mm^3$ and/or white blood cell count less than 1,500/$mm^3$). None of the patients in their series had clinically significant hypersplenism and, after operating on nearly 200 patients for variceal hemorrhage, I have personally encountered only three individuals with life-threatening hypersplenism, who required splenectomy as a component of their operation. The controlled trial by Mutchnick and co-workers[8], which failed to show a beneficial effect of the portacaval shunt on hypersplenism, contained no patients with clinically significant hypersplenism and none developed severe thrombocytopenia or leukopenia during the average follow- up of more than five years.

Thus, the presence or absence of hypersplenism should almost never be a factor determining the selection of operation for variceal hemorrhage. Rather, the effect of the operation on frequency of recurrent hemorrhage and duration and quality of life should be the major considerations influencing choice of procedure. Nonselective and selective shunts are more effective than nonshunting operations for prevention of rebleeding[9]. No controlled trial has demonstrated the superiority of one operation over another with respect to duration of survival. Of the seven controlled investigations of the distal splenorenal shunt versus a variety of nonselective shunts, four have shown a significantly lower frequency of post-shunt encephalopathy after the distal splenorenal shunt and three have demonstrated no difference[10]. Two of these trials[11,12] have compared the distal splenorenal shunt to the conventional splenorenal shunt, the two procedures evaluated by Lodge and associates. One study[11] revealed infrequent encephalopathy after both procedures and the distal splenorenal shunt resulted in fewer neuropsychological disturbances than the conventional splenorenal shunt in the other[12].

Based on the controlled data available, I prefer the selective, distal splenorenal shunt to nonselective shunts, including the conventional splenorenal shunt, for surgical management of variceal hemorrhage. Unless hypersplenism is clinically significant in patients with portal hypertension, its presence should not be a determining factor in choice of operation.

Layton F. Rikker,
Department of Surgery,
University of Nebraska Medical Center,
Omaha, Nebraska, USA.

### *References*

1. Lodge, J.P.A., Mavor, A.I.D., Giles, G.R. Does the warren shunt correct hypersplenism? HPB Surgery (this issue).
2. Soper, N.J., Rikkers, L.F. Cirrhosis and hypersplenism: clinical and hemodynamic correlates. *Curr. Surg.* **43** 21, 1986.
3. Dumont, A.E., Amorosi, E., Stahl, W.M. Significance of splenomegaly in patients with hepatic cirrhosis and bleeding esophageal varices. *Ann. Surg.* **171** 522, 1970.
4. Soper, N.J., Rikkers, L.F. Effect of operations for variceal hemorrhage on hypersplenism. *Am. J. Surg.* **144** 700, 1982.
5. El-Khishen, M.A. Henderson, J.M. Millikan, W.J. Jr, *et al.* Splenectomy is contraindicated for thrombocytopenia secondary to portal hypertension. *Surg. Gynecol. Obstet.* **160** 233, 1985.
6. Marni, A., Trojsi, C., Belli, L. Distal splenorenal shunt: hemodynamic advantage over total shunt and influence on clinical status, hepatic function and hypersplenism. *Am. J. Surg.* **142** 372, 1981.
7. Hutson, D.G., Zeppa, R., Levi, J.U., *et al.* The effect of the distal splenorenal shunt on hypersplenism. *Ann. Surg.* **185** 605, 1977.
8. Mutchnick, M.G., Lerner, E., Conn, H.O. Effect of portacaval anastomosis on hypersplenism. *Dig. Dis. Sci.* **25** 929, 1980.
9. Rikkers, L.F. Bleeding esophageal varices. *Surg. Clin. North Am.* **67** 475, 1987.
10. Rikkers, L.F. Is the distal splenorenal shunt better? *Hepatology* **8** 1705, 1988.
11. Fischer, J.E., Bower, R.H., Atamian, S., *et al.* Comparison of distal and proximal splenorenal shunts: a randomized prospective trial. *Ann. Surg.* **194** 531, 1981.
12. DaSilva, L.C., Strauss, E., Gayotto, L.C.C., *et al.* A randomized trial for the study of the elective surgical treatment of portal hypertension in mansonic schistosomiasis. *Ann. Surg.* **204** 148, 1986.

